# Relation between Blood Pressure Management and Renal Effects of Sodium-Glucose Cotransporter 2 Inhibitors in Diabetic Patients with Chronic Kidney Disease

**DOI:** 10.1155/2019/9415313

**Published:** 2019-11-03

**Authors:** Kazuo Kobayashi, Masao Toyoda, Noriko Kaneyama, Nobuo Hatori, Takayuki Furuki, Hiroyuki Sakai, Masahiro Takihata, Tomoya Umezono, Shun Ito, Daisuke Suzuki, Hiroshi Takeda, Akira Kanamori, Hisakazu Degawa, Hareaki Yamamoto, Hideo Machimura, Atsuko Mokubo, Keiichi Chin, Mitsuo Obana, Toshimasa Hishiki, Kouta Aoyama, Shinichi Nakajima, Shinichi Umezawa, Hidetoshi Shimura, Togo Aoyama, Masaaki Miyakawa

**Affiliations:** ^1^Committee of Hypertension and Kidney Disease, Kanagawa Physicians Association, Yokohama, Kanagawa Prefecture, Japan; ^2^Division of Nephrology, Endocrinology and Metabolism, Department of Internal Medicine, Tokai University School of Medicine, Isehara, Kanagawa 259-1193, Japan

## Abstract

**Aim:**

The renoprotective effect of sodium-glucose cotransporter 2 inhibitors is thought to be due, at least in part, to a decrease in blood pressure. The aim of this study was to determine the renal effects of these inhibitors in low blood pressure patients and the dependence of such effect on blood pressure management status.

**Methods:**

The subjects of this retrospective study were 740 patients with type 2 diabetes mellitus and chronic kidney disease who had been managed at the clinical facilities of the Kanagawa Physicians Association. Data on blood pressure management status and urinary albumin-creatinine ratio were analyzed before and after treatment.

**Results:**

Changes in the logarithmic value of urinary albumin-creatinine ratio in 327 patients with blood pressure < 130/80 mmHg at the initiation of treatment and in 413 patients with BP above 130/80 mmHg were −0.13 ± 1.05 and −0.24 ± 0.97, respectively. However, there was no significant difference between the two groups by analysis of covariance models after adjustment of the logarithmic value of urinary albumin-creatinine ratio at initiation of treatment. Changes in the logarithmic value of urinary albumin-creatinine ratio in patients with mean blood pressure of <102 mmHg (*n* = 537) and those with ≥102 mmHg (*n* = 203) at the time of the survey were −0.25 ± 1.02 and −0.03 ± 0.97, respectively, and the difference was significant in analysis of covariance models even after adjustment for the logarithmic value of urinary albumin-creatinine ratio at initiation of treatment (*p* < 0.001).

**Conclusion:**

Our results confirmed that blood pressure management status after treatment with SGLT2 inhibitors influences the extent of change in urinary albumin-creatinine ratio. Stricter blood pressure management is needed to allow the renoprotective effects of sodium-glucose cotransporter 2 inhibitors.

## 1. Introduction

Sodium-glucose cotransporter 2 inhibitors (SGLT2i) are new oral glucose-lowering agents, which act by increasing urine glucose excretion through inhibition of SGLT2 present in the renal proximal tubules. The associated loss of calories through the huge amount of urinary glucose excretion leads to body weight (BW) loss. In addition to their direct effects on blood glucose level, SGLT2i have other indirect beneficial effects, such as lowering blood pressure (BP) and improving dyslipidemia and liver dysfunction. These pleiotropic effects have attracted attention and wide use of SGLT2i in the treatment of type 2 diabetes mellitus (T2DM), especially in obese patients.

The results of several large-scale cardiovascular outcome clinical trials on SGLT2i, such as the EMPA-REG OUTCOME trial [1] and CANVAS/CANVAS-R program [2], showed significant improvement in cardiovascular events and even diabetic nephropathy in T2DM patients. Our group also reported previously that SGLT2i reduce the urinary albumin-creatinine ratio (ACR; mg/gCr) in Japanese T2DM patients with chronic kidney disease (CKD) [3]. This mechanism of this effect was not completely understood and presumed to be indirectly related to a decrease in BP. In this regard, little is known at present on the renal effects of SGLT2i in low BP patients and the overall effects of BP management on the clinical outcome of treatment with SGLT2i. The aim of the present retrospective study was to investigate these two issues in adult patients with diabetic nephropathy treated with SGLT2i.

## 2. Material and Methods

### 2.1. Study Design and Study Population

The study was approved by the Human Ethics Committee of Kanagawa Medical Association (#1576, August 23, 2016). The study subjects included all 935 T2DM patients who were registered and visited the clinics of medical facilities of the Kanagawa Physicians Association between November 2016 and March 2017. The inclusion criteria of this study were as follows: T2DM patients (1) aged more than 20 years, (2) who commenced treatment with SGLT2i for the first time at least 4 months before the current study, and (3) who had CKD as defined by the K/DOQI clinical practice guidelines for CKD [4]. These guidelines include >3 months of diagnosis of CKD that was based on one of the following criteria: (i) positivity for markers of kidney damage (albuminuria (ACR > 30 mg/gCr), urine sediment abnormalities, electrolyte and other abnormalities associated with tubular disorders, abnormalities detected by histology, and structural abnormalities detected by imaging), (ii) history of kidney transplantation, or (iii) low glomerular filtration rate (GFR) (estimated GFR (eGFR) < 60 ml/min/1.73 m^2^). The following patients were excluded: (1) type 1 DM, (2) on chronic dialysis, (3) had severe liver dysfunction, severe heart failure, or severe infection, (4) malignancy on terminal stage, (5) pregnant women, (6) irregular use of SGLT2i during the study period based on information included in the medical records, and (7) individuals who indicated intention of opt-out at the time of the survey. The following parameters were recorded both at the time of initiation of SGLT2i treatment and at the time of the survey: age, sex, BW, BP (both systolic (SBP) and diastolic (DBP)), serum Cr, hemoglobin A_1c_ (HbA_1c_), ACR (mg/gCr), and proteinuria (quantitative or qualitative). Based on the above criteria, 67 patients were excluded from the study. Thus, the study included 740 patients who were treated with SGLT2i for a median duration of 15.9 months (range 4-36, i.e., the time from the initiation of SGLT2i therapy to the present study). We collected data based on the observations of the GPs. BP measurements at office were performed at each participating medical office using their own validated cuff oscillometric devices. According to the JSH 2014 guidelines [5], BP at office was measured in a quiet environment after the patient rested for a few minutes in the seated position on a chair with their legs not crossed. When two consecutive measurements were taken 1–2 minutes apart, the average of the two measurements was defined as the BP at office. The eGFR was calculated using the following formula: (eGFR (ml/min/1.73 m^2^) = 194 × age^−0.287^ × serum creatinine^−1.094^ × [0.739 for women]) [6].

The study patients were divided into two groups based on BP management status: the “poorly controlled” group, which included 413 patients whose SBP or DBP at the initiation of SGLT2i treatment was over 130 mmHg or 80 mmHg, respectively, and the “well-controlled” group that comprised 327 patients with BP at the initiation of SGLT2i of less than 130/80 mmHg.

**Figure 1:**
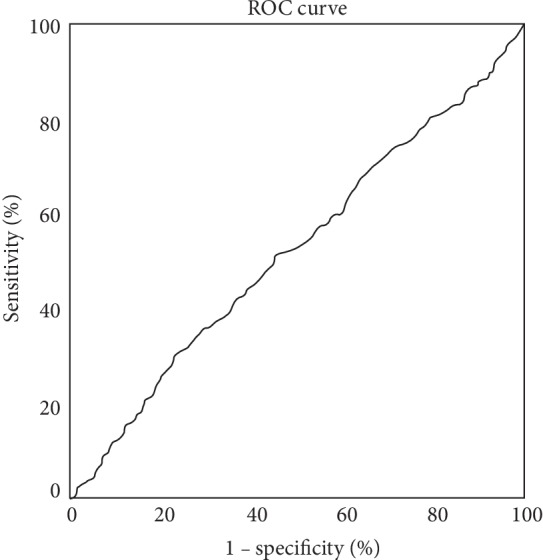
Receiver operating characteristic (ROC) curve showing the overall prediction accuracy of MAP measured at survey and improvement or lack of it in ACR in patients treated with SGLT2 inhibitors.

Based on the receiver operating characteristic (ROC) analysis shown in Figure 1, we also divided the patients into two groups based on mean arterial pressure (MAP; MAP = 1/3 (SBP–DBP) + DBP) at the time of the survey: below 102 mmHg group (*n* = 537) and ≥102 mmHg group (*n* = 203).

The change in the logarithmic value of ACR (*Δ*LNACR) at the end of the treatment period was analyzed retrospectively.

### 2.2. Statistical Analysis

Data that showed normal distribution are reported as mean ± SD while those with skewed distribution are reported as median (lower quartile, upper quartile). Differences between before and after treatment data of the two groups were analyzed by the paired *t*-test for parametric parameters and Wilcoxon signed-rank test for nonparametric parameters, and *p* value less than 0.05 was considered significant. The chi-square test was used to evaluate differences in the percentages of the three groups based on the status of albuminuria.

Analysis of covariance (ANCOVA) was performed with LNACR at the initiation of SGLT2i as the covariate and LNACR at the time of the survey as the dependent variable, taking into consideration BP management at the initiation of SGLT2i treatment (well-controlled versus poorly controlled groups) and the MAP at the time of the survey (<102 versus ≥102 groups).

ROC curve was used to test the overall prediction accuracy of the MAP at the survey and the improvement or lack of improvement in ACR in patients treated with SGLT2i, and the results were reported as the area under the curve (AUC). The IBM SPSS Statistics 25.0 software program (IBM Inc., Armonk, NY) was used in all statistical analyses.

## 3. Results

Supplementary [Supplementary-material supplementary-material-1] lists the background of patients at the time of initiation of SGLT2i treatment, breakdown of SGLT2i, duration of treatment with SGLT2i, and concomitant medications (glucose-lowering agents, antihypertensive agents, and others) at the time of the survey.

The results of ROC analysis showed an estimated optimal cutoff value for a change in MAP (as a marker of improvement) in ACR of 102 mmHg, with sensitivity of 30% and specificity of 23%, with an AUC of 0.52 (95% confidence interval (95% CI) of 0.48-0.56, *p* < 0.0001) (Figure 1).

Table 1 shows the clinical background of patients of the well-controlled and poorly controlled groups at the time of initiation of SGLT2i treatment. BW, BMI, BP at office, eGFR, creatinine clearance rate (CCR), and ACR were significantly higher in patients of the poorly controlled group (*p* < 0.001, *p* < 0.001, *p* < 0.001, *p* = 0.002, *p* < 0.001, and *p* < 0.001, respectively). Patients of the poorly controlled group were significantly younger than those of the well-controlled group (*p* < 0.001).

Table 1 also shows the clinical background of patients of the two MAP groups at the time of the survey. BW, BMI, BP at office, eGFR, CCR, and ACR were significantly higher in the MAP ≥ 102 group than the MAP < 102 group (*p* < 0.001, *p* < 0.001, *p* < 0.001, *p* = 0.018, *p* < 0.001, and *p* = 0.023, respectively). The MAP ≥ 102 group included significantly higher proportion of young males compared with the MAP < 102 group (*p* < 0.001 and *p* = 0.02, respectively).

Table 2 shows changes in LNACR and differences in the clinical findings between the BP well-controlled and poorly controlled groups at the time of initiation of SGLT2i treatment. Changes in eGFR, CCR, HbA_1c_, and BP at office were significantly larger in patients of the poorly controlled group (*p* = 0.007, *p* = 0.016, *p* = 0.023, and *p* < 0.001, respectively). However, there was no significant difference in the changes in LNACR between these two groups.

Table 2 also shows changes in LNACR and differences in the clinical findings between the MAP < 102 group and the MAP ≥ 102 group at the time of the survey. Changes in BW and BP at office were significantly smaller in patients of the MAP ≥ 102 group compared with the MAP < 102 group (*p* = 0.004 and *p* < 0.001, respectively). There was a significant difference in changes in LNACR between these two groups.

**Figure 2:**
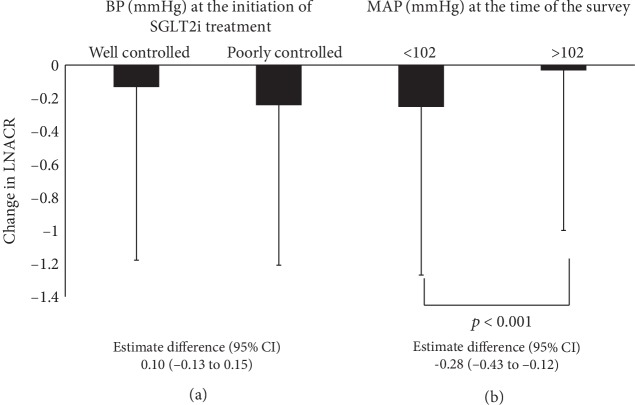
Comparison of changes in urinary albumin-creatinine ratio (ACR) at the initiation of treatment with sodium-glucose cotransporter 2 inhibitors (SGLT2i) and at the time of the survey. Comparisons between patients with BP less than and above 130/80 mmHg at the initiation of SGLT2i (a) and between patients with mean arterial pressure (MAP) < 102 and MAP ≥ 102 mmHg at the time of the survey. Data are mean ± SD. ANCOVA models were adjusted for the LNACR at the initiation of SGLT2i treatment. ANCOVA: analysis of covariance; BP: blood pressure; CI: confidence interval; LNACR: logarithmic value of urinary albumin-creatinine ratio; MAP: mean arterial pressure; SGLT2i: sodium-glucose cotransporter 2 inhibitors.

Figure 2 shows the results of analyses of ANCOVA models after adjustments for LNACR at the initiation of SGLT2i treatment. There was no significant difference in the changes in adjusted LNACR between the BP well-controlled and poorly controlled groups at the time of initiation of SGLT2i treatment (estimated difference (95% CI) was 0.10 (-0.13 to 0.15)). On the other hand, there was a significant difference in the changes in adjusted LNACR between the MAP < 102 and MAP ≥ 102 groups at the time of the survey (*p* < 0.001, estimated difference (95% CI) was -0.28 (-0.43 to 0.12)).

## 4. Discussion

Several cardiovascular outcome clinical trials (CVOCT) of new glucose-lowering agents have been performed to determine their effects on cardiovascular events. The EMPA-REG OUTCOME trial [1] and CANVAS/CANVAS-R program [2], which used SGLT2i, showed the benefits of these agents in reducing cardiovascular events. The TECOS study [7], EXAMINE study [8], and SAVOR-TIMI 53 study [9], which tested DPP4 inhibitors, reported the lack of effect of these agents on cardiovascular events. Patients treated with glucose-lowering agents in these CVOCT had lower level of HbA_1c_ relative to the placebo group, but the appropriate treatment was used in both groups of patients for the management of complications, such as hypertension, <130/80 mmHg, and/or lipid disorders.

What are the mechanisms of the organ protective effects of SGLT2i? Several studies hinted to a multitude of factors, such as body weight loss, improvement of glucose control, correction of dyslipidemia, and lowering of BP. Terami et al. [10] reported that dapagliflozin reduced oxidative stress in rats with diabetic nephropathy while Ferrannini et al. [11] reported the importance of ketone body metabolism in cardiovascular protection. However, Heerspink et al. [12] indicated that the ACR-lowering rate and the direct effect of dapagliflozin itself were the major factors involved in the reduction of ACR in T2DM patients.

We reported recently the renoprotective effect of SGLT2i in Japanese T2DM patients with CKD [3]. The multiregression analysis applied in that study demonstrated the importance of a decrease in BP as an independent factor for the improvement of ACR. We speculated that improvement in ACR in hypertensive patients might be larger than that in normotensive or well-controlled hypertensive patients before SGLT2i treatment. Based on the results of the present study, SGLT2i treatment resulted in a significant improvement in ACR not only in normotensives and well-controlled hypertensive patients but also in poorly controlled hypertensive patients before SGLT2i treatment. Considering our findings together with the above studies, it seems that these improvement effects of ACR by SGLT2i treatment cannot be explained simply by their BP-lowering effect. In fact, the effects observed in our study were similar to the results of the INNOVATION study [13], which showed improvement in ACR by telmisartan in both hypertensive and normotensive Japanese T2DM patients. In this regard, Cherney et al. [14] reported that the changes in ACR by empagliflozin were independent of changes in SBP. Although the changes in ACR by empagliflozin in normotensive patients were not mentioned in their study, our present results were considered to be consistent with their results.

On the other hand, our study showed no significant improvement in ACR in patients of the MAP ≥ 102 group (patients with SBP/DBP of 135/85 mmHg) after treatment with SGLT2i. The pathogenesis of diabetic nephropathy is suggested to involve glomerular hyperfiltration [15]. Hyperfiltration induces activation of tubuloglomerular feedback and the renin-angiotensin-aldosterone system, and these mechanisms play an important role in the progression of diabetic nephropathy [16]. In this regard, previous studies reported that empagliflozin inhibits glomerular hyperfiltration in patients with type 1 DM [17, 18]. While we could not confirm the state of hyperfiltration in hypertensive patients, improvement in hyperfiltration might not be enough to produce a renoprotective effect in the poorly controlled hypertensive patients, as shown in the present study.

The control of BP is an important aspect of the overall management of DM, especially in CKD patients [19]. In most CVOCT, e.g., the EMPA-REG OUTCOME trial [1], CANVAS/CANVAS-R program [2], DECLARE-TIMI 58 study [20], TECOS study [7], EXAMINE study [8], and SAVOR-TIMI 53 study [9], the registered patients received standard care for treatment of DM and cardiovascular risk factors, and in many cases, the target BP was set below 130/80 mmHg throughout the study period. In the TECOS study, 86% of the patients treated with sitagliptin were hypertensives with SBP/DBP at baseline of 135 ± 17/77 ± 10 mmHg [7]. Furthermore, 83% of patients of the EXAMINE study were hypertensives [8], and 82% of those of the SAVOR-TIMI 53 study had hypertension [9]. These data indicate that tight BP control was not achieved in these patients at the time of study entry. Importantly, CVOCT on DPP4 inhibitors reported no clear changes in BP, suggesting that these agents have no benefit in achieving the target BP control. In contrast, BP at the time of the initiation of CVOCT using SGLT2i was 135 ± 17/77 ± 10 mmHg in the empagliflozin group of the EMPA-REG OUTCOME trial (95% of patients received antihypertensive agents) [1], 136 ± 16/78 ± 10 mmHg in the canagliflozin group of the CANVAS/CANVAS-R program (90% were hypertensives) [2], and 135 ± 16 mmHg in all participants of the DECLARE-TIMI study [20]. These values were similar to those of CVOCT using DPP4 inhibitors. However, clear falls in BP were observed in the CVOCT using SGLT2i, suggesting the benefits of these agents in the achievement of target BP control. In the EMPA-REG OUTCOME trial, the frequency of use of antihypertensive agents increased during the study period and no adverse effect of hypotension was observed despite the decrease in BP by SGLT2i [1]. These results highlight the difficulty of management of BP in such patients and that SGLT2i seem useful for BP management. We reported previously that treatment with SGLT2 significantly changed the rates of achieving the target BP (to 130/80 mmHg measured at the office and to 125/75 mmHg in the morning measured at home) in patients with diabetic nephropathy from 26.9% and 25.3% to 34.6% and 34.3%, respectively (*p* < 0.05, each) [21]. A similar decrease in BP was observed in CVOCT using semaglutide (SUSTAIN-6 study [22]). SGLT2i or GLP1 receptor agonists are recommended for hypertensive DM patients because they enhance the achievement rate to target BP relative to DPP4 inhibitors.

The above findings highlight the advantage of using SGLT2i in the dual management of T2DM and BP. It should be noted, however, that our study showed that SGLT2i treatment did not significantly improve ACR in patients with poor BP control. It is possible, however, for patients with poor BP control during treatment with SGLT2i to exhibit improvement in ACR than patients on placebo. Based on the renal data of the EMPA-REG OUTCOME trial [14], ACR decreased from baseline in patients with microalbuminuria and macroalbuminuria, but the relation between ACR improvement and BP control was not discussed in that study. Further studies are needed to investigate the renoprotective effect of SGLT2i in normotensive DM patients or patients with CKD unrelated to diabetic nephropathy.

### 4.1. Limitation

The present study was a retrospective observational study. The use of other glucose-lowering and antihypertensive agents was only investigated at the survey. Patients of the adequate BP group might include those who discontinued antihypertensive agents after the initiation of SGLT2i therapy. Further, our study is only a single-arm study that did not include patients treated with placebo. Although these clinical and methodological limitations do not allow firm conclusions, the large number of patients included in this study provides clear evidence for the benefits of SGLT2i in hypertensive T2DM patients.

## 5. Conclusion

Our results confirmed that blood pressure management status after SGLT2i administration influences the extent of change in urinary albumin-creatinine ratio. Stricter BP management might be needed in general practice to demonstrate the renoprotective effects of SGLT2i in Japanese T2DM patients with CKD.

## Figures and Tables

**Table 1:** Differences among the study groups based on blood pressure at initiation of treatment and MAP at the time of survey.

	BP (mmHg) at initiation of SGLT2i treatment		
	Well-controlled group (*n* = 327)	Poorly controlled group (*n* = 413)	*p* value
Age (years)	62.2 ± 12.4	58.7 ± 12.6	<0.001
Sex, males : females	210 : 117	273 : 140	n.s.
BW (kg)	73.6 ± 15.0	79.0 ± 17.3	<0.001
BMI (kg/m^2^)	26.6 ± 4.7	28.1 ± 5.2	<0.001
SBP/DBP at office (mmHg)	124 ± 12.3/69.0 ± 8.5	149 ± 16.6/86.5 ± 10.0	<0.001/<0.001
MAP at office (mmHg)	87.4 ± 7.3	107.3 ± 9.8	<0.001
HbA_1c_ (mmol/mol) (%)	62.5 ± 16.1 (7.9 ± 1.5)	63.9 ± 16.4 (8.0 ± 1.5)	n.s.
eGFR (ml/min/1.7 m^2^)	75.9 ± 23.8	81.4 ± 23.7	0.002
CCR (ml/min)	106 ± 48.3	123 ± 53.5	<0.001
ACR (mg/gCr)	38.1 (13.3, 103.6)	58.5 (24.0, 185.9)	<0.001
Logarithmic value of ACR	3.77 ± 1.51	4.25 ± 1.58	<0.001
ACR < 30/30‐300/≥300 mg/gCr, *n*	125/167/35	114/224/75	<0.001
Duration of treatment (months)	15 (11, 22)	12 (10, 24)	n.s.

	MAP (mmHg) at the time of the survey		
	<102 (*n* = 537)	≥102 (*n* = 203)	*p* value
Age (years)	61.5 ± 12.6	57.0 ± 11.8	<0.001
Sex, males : females	337 : 200	146 : 57	0.02
BW (kg)	75.1 ± 16.1	80.6 ± 17.0	<0.001
BMI (kg/m^2^)	26.9 ± 4.8	28.8 ± 5.4	<0.001
SBP/DBP at office (mmHg)	135 ± 18.1/75.9 ± 11.6	147 ± 19.6/86.5 ± 12.4	<0.001/<0.001
MAP at office (mmHg)	95.4 ± 11.9	106.7 ± 13.1	<0.001
HbA_1c_ (mmol/mol) (%)	63.0 ± 16.3 (7.9 ± 1.5)	64.3 ± 16.3 (8.0 ± 1.5)	n.s.
eGFR (ml/min/1.7 m^2^)	77.7 ± 24.0	82.4 ± 23.5	0.018
CCR (ml/min)	111 ± 50.9	127 ± 52.9	<0.001
ACR (mg/gCr)	42.6 (16.2, 140.0)	58.5 (26.1, 164.9)	0.023
Logarithmic value of ACR	3.96 ± 1.56	4.24 ± 1.55	0.027
ACR < 30/30‐300/≥300 mg/gCr, *n*	185/275/77	54/116/33	n.s.
Duration of treatment (months)	15 (11, 23)	12 (8, 24)	n.s.

^∗^ *p* < 0.05, ^¶^*p* < 0.01, compared with the other group (in two-group comparisons); ^§^*p* < 0.01, compared with the <125/75 group; and ^†^*p* < 0.01, compared with the ≥130/80 to <135/85 group. Abbreviations: ACR: urinary albumin-creatinine ratio; BMI: body mass index; BW: body weight; SBP: systolic blood pressure; DBP: diastolic blood pressure; CCR: creatinine clearance, calculated by the Cockcroft-Gault formula; eGFR: estimated glomerular filtration rate; HbA_1c_: hemoglobin A_1c_; MAP: mean arterial pressure; n.s.: not significant; SGLT2i: sodium-glucose cotransporter 2 inhibitors.

**Table 2:** Delta changes in parameters of blood pressure and diabetes control.

	BP (mmHg) at initiation of SGLT2i treatment		
	Well-controlled group (*n* = 327)	Poorly controlled group (*n* = 413)	*p* value
*Δ*logarithmic value of ACR	−0.13 ± 1.05	−0.24 ± 0.97	n.s.
*Δ*eGFR (ml/min/1.73 m^2^)	−1.8 ± 10.9	−4.0 ± 11.2	0.007
*Δ*CCR (ml/min)	−6.0 ± 14.7	−9.0 ± 17.7	0.016
*Δ*HbA_1c_ (mmol/mol) (%)	−6.2 ± 12.6	−8.5 ± 14.7	0.023
*Δ*BW (kg)	−2.4 ± 3.6	−2.7 ± 4.4	n.s.
*Δ*SBP/*Δ*DBP at office (mmHg)	2.6 ± 15.7/3.5 ± 10.0	−12.8 ± 18.3/−5.6 ± 10.2	<0.001/<0.001
*Δ*MAP at office (mmHg)	3.2 ± 10.3	−8.0 ± 11.2	<0.001

	MAP (mmHg) at the time of the survey		
	<102 (*n* = 537)	≥102 (*n* = 203)	*p* value
*Δ*logarithmic value of ACR	−0.25 ± 1.02	−0.03 ± 0.97	<0.001
*Δ*eGFR (ml/min/1.73 m^2^)	−3.2 ± 11.0	−2.7 ± 11.4	n.s.
*Δ*CCR (ml/min)	−8.3 ± 15.4	−5.9 ± 18.4	n.s.
*Δ*HbA_1c_ (mmol/mol) (%)	−7.4 ± 13.8	−7.8 ± 14.0	n.s.
*Δ*BW (kg)	−2.8 ± 3.7	−1.9 ± 4.8	0.004
*Δ*SBP/*Δ*DBP at office (mmHg)	−8.7 ± 17.7/−3.2 ± 10.5	1.2 ± 19.9/2.8 ± 11.4	<0.001/<0.001
*Δ*MAP at office (mmHg)	−5.0 ± 11.4	2.3 ± 12.6	<0.001

Abbreviations: ACR: urinary albumin-creatinine ratio; BW: body weight; CCR: creatinine clearance, calculated by the Cockcroft-Gault formula; DBP: diastolic blood pressure; *Δ*: change in; eGFR: estimated glomerular filtration rate; HbA_1c_: hemoglobin A_1c_; MAP: mean arterial pressure; n.s.: not significant; SGLT2i: sodium-glucose cotransporter 2 inhibitors; SBP: systolic blood pressure.

## Data Availability

The data used to support the findings of this study are available from the corresponding author upon request.

## Abbreviations

ACR: Urinary albumin-creatinine ratio
ANCOVA: Analysis of covariance
BP: Blood pressure
BW: Body weight
BMI: Body mass index
CI: Confidence interval
CKD: Chronic kidney disease
DPP4: Dipeptidyl peptidase-4
DBP: Diastolic blood pressure
*Δ*ACR: Change in urinary albumin-creatinine ratio
eGFR: Estimated glomerular filtration rate
GP: General practitioner
GLA: Glucose-lowering agents
GLP1: Glucagon-like peptide-1
HbA_1c_: Hemoglobin A_1c_
LNACR: Logarithmic value of urinary albumin-creatinine ratio
MAP: Mean arterial pressure
RAS: Renin-angiotensin system
ROC: Receiver operating characteristic
SBP: Systolic blood pressure
SGLT2i: Sodium-glucose cotransporter 2 inhibitors
T2DM: Type 2 diabetes mellitus.

## Supplementary Materials

Supplementary Materials The backgrounds of patients at the time of initiation of SGLT2i treatment; age, sex, BW, BMI, BP, HbA_1c_, eGFR, CCR, and ACR are shown in Supplementary Table 1: six kinds of SGLT2i, ipragliflozin, dapagliflozin, tofogliflozin, luseogliflozin, canagliflozin, and empagliflozin, are used in the patients of this study. The duration of administration of SGLT2i is divided into 4 groups (from 4 to 12 months, from 13 to 18 months, from 19 to 24 months, and over 25 months), and the number of patients in each groups is shown in this table. The concomitant medications (glucose-lowering agents, antihypertensive agents, and statins) at survey time are also shown in this table.
