# Supplementary material for: Relation between Blood Pressure Management and Renal Effects of Sodium-Glucose Cotransporter 2 Inhibitors in Diabetic Patients with Chronic Kidney Disease
Source: J Diabetes Res. 2019 Nov 3;2019:9415313. doi: 10.1155/2019/9415313 (PMC6875192; doi:10.1155/2019/9415313)
Supplement: Supplementary Materials — The backgrounds of patients at the time of initiation of SGLT2i treatment; age, sex, BW, BMI, BP, HbA1c, eGFR, CCR, and ACR are shown in Supplementary Table 1: six kinds of SGLT2i, ipragliflozin, dapagliflozin, tofogliflozin, luseogliflozin, canagliflozin, and empagliflozin, are used in the patients of this study. The duration of administration of SGLT2i is divided into 4 groups (from 4 to 12 months, from 13 to 18 months, from 19 to 24 months, and over 25 months), and the number of patients in each groups is shown in this table. The concomitant medications (glucose-lowering agents, antihypertensive agents, and statins) at survey time are also shown in this table. [file 9415313.f1.docx]

Supplementary Table 1. Clinical background of study patients.

| Background of patients at the time of initiation of SGLT2i treatment | |
| --- | --- |
| Age, years | 60.2±12.6 (21-97) |
| Sex, male:female | 483:257 |
| BW, kg | 76.6±16.5 |
| BMI, kg/m^2^ | 27.4±5.0 |
| SBP/DBP/MAP at office, mmHg | 138±19/79±13/99±13 |
| HbA_1c_, mmol/mol (%) | 63.3±16.3 (7.9±1.5%) |
| eGFR, mL/min/1.7 m^2^ | 79.0±23.9 |
| CCR, mL/min | 115.4±52.0 |
| ACR, mg/gCr | 46.8 [17.8, 148.0] |
| ACR <30/30 to <300/≥300 mg/gCr, n | 239/391/110 |
| SGLT2i (in order of release in Japan)* | n=740, n (daily dose, percentage of patients) |
| Ipragliflozin | 237 (25 mg;7%, 50 mg; 91%, 100 mg; 2%) |
| Dapagliflozin | 128 (2.5 mg; 1%, 5 mg; 93%, 10 mg; 6%) |
| Tofogliflozin | 110 (10 mg; 15%, 20 mg; 85%) |
| Luseogliflozin | 60 (2.5 mg; 87%, 5 mg; 13%) |
| Canagliflozin | 80, all 100 mg |
| Empagliflozin | 123 (10 mg; 97%, 25 mg; 3%) |
| Duration of administration of SGLT2i, months | 14.0 [10.0, 24.0], Average; 14.8 |
| 4-12 | 334 |
| 13-18 | 137 |
| 19-24 | 206 |
| >25 | 63 |
| Medications used at survey time, n (% of patients) | Average; 2.1 drugs |
| Glucose lowering agents, n (% of patients) |  |
| SGLT2i alone | 69 (9.3%) |
| DPP4 inhibitors | 418 (56.5%) |
| Sulphonylureas | 244 (33.0%) |
| Metformin | 432 (58.4%) |
| Insulin | 190 (25.7%) |
| GLP-1 receptor agonists | 95 (12.8%) |
| Pioglitazone | 108 (14.6%) |
| Others (αGI, glinides etc) | 66 (8.9%) |
| Antihypertensive agents, n (% of patients) | (average; 1 drug) |
| none | 315 (38.0%) |
| renin-angiotensin-aldosterone system inhibitors | 393 (53.1%) |
| Ca channel blockers | 214 (28.9%) |
| Aldosterone blockers | 20 (2.7%) |
| Diuretics (thiazides) | 26 (3.5%) |
| Diuretics (loops) | 24 (3.2%) |
| β-blockers | 40 (5.4%) |
| Others (α-blockers, etc.) | 18 (1.6%) |
| Other medications |  |
| Statins | 379 (51.2%) |

* SGLT2i was changed during the administration period in 2 cases.

Abbreviations as in supplementary table 1; ACR; urinary albumin creatinine ratio, BMI; body mass index, BW; body weight, CCR; creatinine clearance, calculated by Cockcroft-Gault formula, DBP; diastolic blood pressure, DPP-4; dipeptidyl peptidase-4, eGFR: estimated glomerular filtration rate, GLP-1; Glucagon-like peptide-1, HbA_1c_; hemoglobin A_1c_, MAP; mean arterial pressure, SGLT2i; sodium-glucose co-transporter 2 inhibitor, SBP; systolic blood pressure,αGI; alpha glucosidase inhibitor **.**

**Supplementary table legend;**

The backgrounds of patients at the time of initiation of SGLT2i treatment; age, sex, BW, BMI, BP, HbA_1c,_ eGFR, CCR and ACR are shown in supplementary table 1. Six kinds of SGLT2i; ipragliflozin, dapagliflozin, tofogliflozin, luseogliflozin, canagliflozin and empagliflozin, are used in the patients of this study.The duration of administration of SGLT2i are divided into 4 groups (from 4 to 12 months, from 13 to 18 months, from 19 to 24 months and over 25 months) and the number of patients in each groups are shown in this table. The concomitant medications (glucose lowering agents, antihypertensive agents and statins) at survey time are also shown in this table.
